# Oral health status and oral health-related quality of life among patients with type 2 diabetes mellitus in the United Arab Emirates: a matched case-control study

**DOI:** 10.1186/s12955-020-01418-9

**Published:** 2020-06-15

**Authors:** Nadia Khalifa, Betul Rahman, Marianna D. Gaintantzopoulou, Suhail Al-Amad, Manal M. Awad

**Affiliations:** 1 Department of Preventive and Restorative Dentistry, College of Dental Medicine, University of Sharjah, Sharjah, UAE; 2 Department of Biomaterials, School of Dentistry, National and Kapodistrian University of Athens, Athens, Greece; 3 Department of Oral and Craniofacial Health Sciences, College of Dental Medicine, University of Sharjah, Sharjah, UAE; 4 College of Dental Medicine, University of Sharjah, Sharjah, UAE

**Keywords:** Clinical attachment loss, DMFT, OHIP-14, Type 2 diabetes mellitus, UAE

## Abstract

**Background:**

Nearly a quarter of the population in the UAE has type 2 diabetes mellitus (T2DM), and this medical condition is associated with poorer oral health. The effects on oral health-related quality of life (OHRQoL), however, have not been examined in this population. Therefore, the objective of this study was to assess the impact of oral health problems, such as caries and periodontitis, on OHRQoL among Arab patients with and without T2DM.

**Methods:**

This matched case-control study included 88 diabetic and 88 non-diabetic participants recruited from University Dental Hospital Sharjah and University Hospital Sharjah, UAE. Participants completed a sociodemographic questionnaire as well as the Oral Health Impact Profile short form (OHIP-14), which measures OHRQoL. Clinical examinations were conducted to assess participants’ dental caries status, using the Decayed Missing Filled Teeth (DMFT) Index, and periodontal condition, via clinical attachment loss (CAL) dichotomized to CAL < 3 mm and CAL ≥3 mm. Linear regression models were used to identify the association among OHIP domains, clinical attachment loss, DMFT scores, and diabetes status.

**Results:**

The mean age of participants was 43.0 years. A significantly (*p* = 0.01) higher proportion of diabetic patients (23%) had a CAL ≥3 mm than non-diabetic patients (10%). No significant differences in OHIP scores were observed between diabetic and non-diabetic patients. The results of the linear regression suggested that irrespective of diabetic status, DMFT scores were significantly associated with physical disability, physical pain, psychological discomfort, and psychological disability, as well as total OHIP scores. CAL was significantly associated with the handicap domain. Among non-diabetic patients, OHIP scores were significantly associated with DMFT scores in five OHIP domains (functional limitation, physical disability, physical pain, psychological discomfort, psychological disability), as well as total OHIP scores. Among diabetic patients, CAL was significantly associated with both the social disability and handicap domains, while only the handicap domain reached statistical significance among non-diabetic patients.

**Conclusions:**

Participants who had decayed teeth, irrespective of their diabetic status, reported substantial physical and psychological impacts on OHRQoL. CAL also had a significant impact on OHRQoL, being primarily associated with the OHIP handicap domain in both diabetic and non-diabetic patients.

## Background

The current concept of health emphasizes not only the absence of disease, but also a complete state of well-being. As such, the patients’ experiences and perceptions of their own conditions must be taken into consideration by measuring their health-related quality of life (HRQoL) [1].

Diabetes is a chronic metabolic disease characterized by hyperglycemia caused by a defect in insulin secretion, insulin function, or both [2]. Globally, it was estimated that in 2017, 451 million people aged 18–99 years had diabetes, and that these figures are expected to increase to 693 million by 2045 [3]. The UAE has one of the highest prevalence rates of type 2 diabetes (T2DM), with a crude prevalence rate of 20% [4].

Oral health problems such as periodontal disease, dental caries, and tooth loss can negatively affect patients’ HRQoL [5–8]. Diabetes may be associated with oral complications such as xerostomia, taste impairment, oral candidiasis, and periodontal disease [9–12]. Patients with poorly controlled or uncontrolled diabetes are especially susceptible to periodontal disease, which is characterized by progressive alveolar bone loss, clinical attachment loss (CAL), and deepened periodontal pockets [13]. Advanced glycation end products, which accumulate in the tissues and plasma of diabetic patients, are considered to be responsible for increased inflammation and exaggerated release of pro-inflammatory cytokines, followed by the destruction of periodontal tissues [14]. Periodontal infection may also have an adverse effect on the metabolic control of diabetes [15]. Some studies have suggested that the prevention and treatment of periodontal disease may contribute to an improvement in metabolic control of diabetes, and should thus be considered an integral part of diabetes management [16].

One of the most common complaints of diabetic patients is xerostomia, which can contribute to oral problems such as the occurrence of tooth decay, especially root caries [17]. These may ultimately lead to oral pain, dysfunction, and a reduction in overall well-being and quality of life. Such oral health problems are only expected to increase in the upcoming decades, given increasing trends in the prevalence of T2DM and the retention of teeth into older age.

There are different methods of measuring oral and HRQoL in specific population groups, such as patients with diabetes. The most popular method is the use of multiple-item, standardized questionnaires that capture the subject’s perception of their own physical and psychological health, as well as function. There are inconsistent findings in the literature investigating the association between diabetes, oral health status, and quality of life. In a recent review, Cervino et al. [18] established the existence of a correlation between oral health and quality of life among diabetic patients. This is in contrast to other studies that did not observe an impact of type 2 diabetes on oral health-related quality of life (OHRQoL), as measured using the Oral Health Impact Profile-49 (OHIP-49) [8] and OHIP-20 questionnaires [6, 19].

Another study compared the discriminative capability of the Persian versions of the Geriatric/General Oral Health Assessment Index (GOHAI) and OHIP-14 questionnaires among diabetic and non-diabetic patients [5]. In this study, the oral health status of diabetic patients was poor, and oral problems affected oral health-related quality of life. The authors also reported that the OHIP-14 had higher discriminatory abilities and was more effective in diagnosing oral problems [5]. Sandberg et al. used the 36-item Short Form Health Survey (SF-36) to assess HRQoL in diabetic patients and healthy controls, and found that diabetic patients exhibited less favorable scores in the domains of physical functioning, role functioning-physical, general health, and social functioning, when compared to controls [20].

Therefore, the objective of this study was to assess the impact of oral health problems, such as caries and periodontitis, on HRQoL among Arab patients with and without T2DM, with the overarching aim of informing the development of oral health care treatments and preventive measures that are tailored to the needs of this medically compromised patient group.

## Methods

This case-control study was carried out at the University Dental Hospital Sharjah and University Hospital Sharjah, where a sample of adults with T2DM were selected from a list of patients attending the clinics between March 2016 and May 2018. The diagnosis of T2DM was based on subjects’ existing medical records, and additional inclusion criteria comprised of the following: (1) at least 10 natural teeth; (2) no known major medical complications such as coronary heart disease; (3) no antibiotic use during the last 3 months; (4) no steroidal or non-steroidal anti-inflammatory medication use during the last 3 weeks; (5) no treatment with immunosuppressive chemotherapy; (6) no professional periodontal treatment received during the last 6 months; and (7) no pregnancy or lactation. Age-matched non-diabetic individuals, registered at the same hospitals, were enrolled into the control group. To confirm that participants in the control group were not diabetic, fasting blood glucose levels were checked and those with values above 110 mg/dl were excluded.

The sample size, consisting of 176 participants, was calculated based on a previous study [7]. It was estimated that, by maintaining 90% power and using a two-sided alpha level of 0.05, approximately 168 participants (84 diabetic and 84 non-diabetic patients) would be needed to observe a statistically and clinically important difference of 4 points on the OHIP-14 scores (SD: 8) between diabetic and non-diabetic participants. To compensate for possible refusal/drop-out of participants, a total of 88 subjects were recruited to each group.

Patients were first asked to complete a short questionnaire pertaining to sociodemographic characteristics. They were then asked to complete the OHIP-14, a self-administered questionnaire that measures HRQoL using 14 items that assess seven domains: functional limitation, physical pain, psychological discomfort, physical disability, psychological disability, social disability, and handicap. Each domain is assessed by two questions that ask subjects how frequently they had experienced specific negative impacts during the preceding 12 months. Responses to the items are recorded by using a five-point Likert scale (0, never; 1, hardly ever; 2, occasionally; 3, fairly often; 4, very often). The overall score for the OHIP-14 is obtained by summing all responses, and ranges from 0 to 56 points [21].

The reliability, validity, responsiveness, and discriminant abilities of this instrument have been previously established in several clinical trials and observational studies [22, 23]. The Arabic version of this questionnaire was previously tested and validated in Sudan [23], Jordan [7], Saudi Arabia [24], and the United Arab Emirates [25].

The study protocol was reviewed and approved by the University of Sharjah Research Ethics Committee (Approval No: ERC/23/11/15/45), and written consent was obtained from the participants prior to taking part in the study.

### Clinical examination

Two dentists were trained by a senior specialist on the clinical indices to ensure good intra- and inter-examiner reliability (intraclass correlation coefficient value ≥0.75). The dentists were blinded to the participants’ diabetic status and conducted a full-mouth clinical periodontal examination at six sites per tooth (third molars excluded), using a manual periodontal probe with Williams markings and a tip diameter of 0.45 mm. The oral examinations were carried out on portable dental chairs at the University Hospital Sharjah, or conventional dental chairs at the University Dental Hospital Sharjah. The periodontal examination included assessments of the pocket depth (the distance from the free gingival margin to the base of the probeable pocket, recorded to the nearest mm) and gingival recession (location of the free gingival margin in relation to the cemento-enamel junction). The latter was determined to be positive if located apical to the cemento-enamel junction, and negative if located coronal to the cemento-enamel junction. The algebraic sum of the pocket depth and gingival recession was used to compute the CAL. The Decayed Missing Filled Teeth (DMFT) Index score was obtained by summing up the number of decayed, missing, and filled teeth.

### Data analysis

The Statistical Package for Social Sciences (SPSS) Version 26.0 (IBM Corp., Armonk, NY, USA) was used for data processing and analysis. Participant characteristics were described using frequency distribution for categorical variables, and mean and standard deviation for continuous variables. The average clinical attachment levels were dichotomized as CAL < 3 mm and CAL ≥3 mm, and DMFT scores were computed for each individual and then averaged across participants in the groups. OHIP-14 scores were compared between groups using the independent t-test. This analysis supports the hypothesis that type 2 diabetes is as a “risk factor” for worse OHRQoL within the UAE population. Linear regression analyses were conducted to assess the association between the OHIP-14 subscales and potential explanatory variables (i.e., diabetes status, CAL, and DMFT scores).

## Results

In this study, 88 T2DM patients were matched to 88 non-diabetic patients on age, marital status, and level of education. There were significantly (*p* = 0.02) more female diabetic patients. A significantly higher proportion of diabetic patients had a CAL ≥3 mm compared to non-diabetic patients (23% vs. 10%, respectively). No significant differences in mean DMFT scores (Table 1) or OHIP scores (Table 2) were observed between diabetic and non-diabetic patients.

**Table 1:** Characteristics of study participants

Variable		Non-diabetic *N* = 88	Diabetic *N* = 88	*P*-Value
Age, mean (SD)		43.0 (1.5)	43.1 (1.5)^a^	0.98
Duration of diabetes in years		NA	7.3 (6.4)	NA
Sex	Males (%)	48 (55)	32 (36)^b^	0.02
	Females (%)	40 (45)	56 (64)	
Marital status	Single (%)	25 (28)	25 (28)^b^	1.0
	Married (%)	63 (72)	63 (72)	
Level of education	School (%)	32 (36)	32 (37)^b^	0.96
	University (%)	56 (64)	55 (63)	
Smoking	No (%)	65 (75)	63 (72)^b^	0.61
	Yes (%)	22 (25)	25 (28)	
Clinical attachment loss	CAL < 3 mm	80 (90)	68 (77)^b^	0.01
	CAL ≥ 3 mm	8 (10)	20 (23)	
Decayed (D) Missing (M) Filled (F) Teeth	D (SD)	4.5 (4.9)	3.8 (4.3)^a^	0.37
	M (SD)	3.4 (4.6)	3.3 (4.6)^a^	0.97
	F (SD)	5.0 (5.2)	5.1 (4.9)^a^	0.92

Numbers do not add up to the full sample size due to missing individual data

^a^Based on independent t-test; ^b^Based on Chi-square test

**Table 2:** Association between OHIP-14 domains and diabetes status

Domain	Non-diabetic Mean (SD)	Diabetic Mean (SD)	*P*-value*
Functional Limitations *(trouble pronouncing words/worsened taste)*	1.2 (1.6)	1.5 (1.4)	0.2
Physical Disability *(interrupted meals/poor diet)*	2.6 (1.9)	2.7 (1.8)	0.6
Physical Pain *(oral pain/discomfort while eating)*	2.4 (2.0)	2.5 (2.1)	0.6
Psychological Discomfort *(feeling tense/self- conscious)*	2.0 (1.9)	2.2 (1.9)	0.72
Psychological Disability *(difficulty relaxing/embarrassment)*	1.7 (1.7)	1.9 (1.7)	0.38
Social Disability *(irritability/difficulty doing usual jobs)*	1.2 (1.4)	1.5 (1.6)	0.17
Handicap *(life less satisfying/inability to function)*	1.4 (1.5)	1.3 (1.6)	0.63
Total OHIP	12.4 (9.5)	13.7 (9.7)	0.37

*Based on independent t-test; *OHIP* Oral Health Impact Profile

Table 3 shows the linear regression model for the association between OHIP-14 domains and clinical variables (CAL, DMFT scores), and diabetes status. Irrespective of diabetes status, DMFT scores were significantly (*p* < 0.05) associated with physical disability (*B* = 0.11, 95% confidence intervals [CI]: 0.06, 0.16), physical pain (*B* = 0.06, 95% CI: 0.01,0.11), psychological discomfort (*B* = 0.07, 95% CI: 0.02, 0.10), psychological disability (*B* = 0.06, 95% CI: 0.02, 0.10), as well as total OHIP scores (*B* = 0.42, 95% CI: 0.19, 0.64). CAL was significantly associated with handicap (*B* = 0.84, 95% CI: 0.17, 1.51; Table 3).

**Table 3:** Association of clinical variables and diabetes status with oral health-related quality of life

Domain	Variable	Parameter Estimate^a^	95% Confidence Interval
Functional Limitations	Diabetics/non-diabetics	0.42	0.02, 0.86
	CAL ≥3/CAL < 3	0.07	−0.54, 0.67
	DMFT	0.07	−0.02, 0.11
Physical Disability	Diabetics/non-diabetics	0.34	−0.25, 0.93
	CAL ≥3/CAL < 3	− 0.25	−1.1, 0.56
	DMFT	0.11	0.06, 0.16
Physical Pain	Diabetics/non-diabetics	0.12	−0.48, 0.72
	CAL ≥3/CAL < 3	0.66	−0.17, 1.49
	DMFT	0.06	0.01, 0.11
Psychological Discomfort	Diabetics/non-diabetics	0.12	−0.44, 0.67
	CAL ≥3/CAL < 3	0.53	−1.2, 0.04
	DMFT	0.07	0.02, 0.10
Psychological Disability	Diabetics/non-diabetics	0.31	−0.2, 0.84
	CAL ≥3/CAL < 3	0.03	−0.68, 0.74
	DMFT	0.06	0.02, 0.10
Social Disability	Diabetics/non-diabetics	0.25	−0.20, 0.70
	CAL ≥3/CAL < 3	0.58	−0.05, 1.21
	DMFT	0.01	−0.02, 0.05
Handicap	Diabetics/non-diabetics	0.03	−0.44, 0.51
	CAL ≥3/CAL < 3	0.84	0.17, 1.51
	DMFT	0.02	−0.02, 0.06
Total OHIP	Diabetics/non-diabetics	1.44	−1.36, 4.26
	CAL ≥3/CAL < 3	2.18	−1.70, 6.05
	DMFT	0.42	0.19, 0.64

^a^Linear regression adjusted for gender and marital status

In non-diabetics, DMFT scores were significantly (*p* < 0.05) associated with scores in five OHIP domains, namely, functional limitation (*B* = 0.08, 95% CI: 0.03, 012), physical disability (*B* = 0.09, 95% CI: 0.03, 0.15), physical pain (*B* = 0.13, 95% CI: 0.08, 0.19), psychological discomfort (*B* = 0.07, 95% CI: 0.02, 0.13), and psychological disability (*B* = 0.08, 95% CI: 0.02, 0.13), as well as total OHIP scores (*B* = 0.45, 95% CI: 0.19, 0.74; *p* < 0.05; Table 4).

**Table 4:** Association of clinical variables with oral health-related quality of life among non-diabetics

Domain	Variable	Parameter Estimate^a^	95% Confidence Interval
Functional Limitations	CAL ≥3/CAL < 3	− 0.73	− 1.80, 0.35
	DMFT	0.08	0.03, 0.12
Physical Disability	CAL ≥3/CAL < 3	−0.21	−1.57, 1.16
	DMFT	0.09	0.03, 0.15
Physical Pain	CAL ≥3/CAL < 3	−1.11	−2.4, 0.19
	DMFT	0.13	0.08, 0.19
Psychological Discomfort	CAL ≥3/CAL < 3	− 0.23	− 1.55, 1.09
	DMFT	0.07	0.02, 0.13
Psychological Disability	CAL ≥3/CAL < 3	− 0.58	− 1.78, 0.63
	DMFT	0.08	0.02, 0.13
Social Disability	CAL ≥3/CAL < 3	−0.17	−1.18, 0.83
	DMFT	0.02	−0.02, 0.06
Handicap	CAL ≥3/CAL < 3	−0.04	−1.24, 1.17
	DMFT	−0.01	−0.05, 0.05
Total OHIP	CAL ≥3/CAL < 3	−3.1	−9.28, 3.2
	DMFT	0.45	0.19, 0.74

^a^Linear regression adjusted for gender and marital status

On the other hand, among diabetic patients, CAL was significantly (*p* < 0.05) associated with the social disability (*B* = 0.98, 95% CI: 0.11, 1.88) and handicap (*B* = 1.2, 95% CI: 0.36, 2.01) domains (*p* < 0.05; Table 5).

**Table 5:** Association of clinical variables with oral health-related quality of life among diabetic patients

Domain	Variable	Parameter Estimate^a^	95% Confidence Interval
Functional Limitations	CAL > 3/CAL < 3	0.47	− 0.32, 1.26
	DMFT	0.05	−0.02, 0.1
Physical Disability	CAL > 3/CAL < 3	1.08	− 0.03, 2.11
	DMFT	0.02	−0.05, 0.10
Physical Pain	CAL ≥3/CAL < 3	0.18	− 0.004, 0.15
	DMFT	0.07	−0.88, 1.25
Psychological Discomfort	CAL > 3/CAL < 3	0.99	−0.01, 2.0
	DMFT	0.02	−0.05, 0.10
Psychological Disability	CAL > 3/CAL < 3	0.43	− 0.50, 1.36
	DMFT	0.03	−0.04, 0.10
Social Disability	CAL > 3 /CAL < 3	0.98	0.11, 1.88
	DMFT	−0.01	− 0.41, 1.09
Handicap	CAL > 3/CAL < 3	1.2	0.36, 2.01
	DMFT	−0.1	0.01, 0.11
Total OHIP	CAL > 3/CAL < 3	4.86	−0.27, 9.9
	DMFT	0.23	−0.14, 0.61

^a^Linear regression adjusted for gender and marital status

## Discussion

In this matched case-control study of 88 diabetic and 88 non-diabetic individuals in the UAE, those who had decayed teeth, irrespective of their diabetic status, reported substantial physical and psychological impacts on OHRQoL. In addition to being the first study performed in the UAE to explore the association between diabetes and oral health related quality of life, we used a case matching technique that added validity to the findings of the study. Furthermore, we were able to compare information gathered from the study participants with the hospital medical records, thus reducing the risk of recall bias. Moreover, checking fasting blood glucose levels of the control group so that those with high glucose levels (above 110 mg/dl) could be excluded also added to the validity of the study findings.

Studies have shown that periodontal disease is more prevalent and severe among diabetic patients, compared to healthy individuals [14, 26, 27]. This has been attributed to an increased inflammatory response in periodontal tissues, due to abnormal host responses in diabetic patients [14]. The entrance of periodontal microorganisms and their virulence factors into the circulation may have a role in the elevation of acute phase and oxidative stress biomarkers. Interactions between advanced glycation end products and their receptors, as well as oxidative-stress-mediated pathways, provide possible mechanistic links between diabetes and periodontitis [28]. Indeed, studies have reported associations between poorly controlled diabetes and the continued progression of attachment loss [29].

Periodontitis staging considers the severity of interdental CAL as slight (1–2 mm), moderate (3–4 mm), or severe (5 mm or more). Accordingly, in this study, CAL was dichotomized as < 3 mm (slight attachment loss) and ≥ 3 mm (moderate or severe attachment loss). In agreement with previous studies [26, 27], there was significantly more CAL in diabetic (23%) than non-diabetic (10%) patients in the current study, which provides further evidence that diabetes can increase the occurrence of periodontitis.

The extent of diabetes is considered as a key factor when determining the susceptibility to periodontitis and other systemic complications [30]. Although the mean age (43 years) was similar in both diabetic and non-diabetic patients in this study, diabetic patients had a longer duration of periodontal disease (> 7 years), which might explain the higher prevalence of more severe periodontal disease in them than in non-diabetic patients.

The association between dental caries and diabetes mellitus is controversial [10, 31]. Diabetes is associated with impaired salivary gland function and increased blood and salivary glucose concentrations, which favor oral bacterial growth, thus leading to an increased risk of developing dental caries [31–33]. Despite this possible biological explanation, the duration of diabetes may also be a contributing factor. Our study sample was relatively young, with an average duration of diabetes less than that reported by other studies [34]. This may explain the lack of significant differences in DMFT scores observed between the diabetic and non-diabetic groups.

Likewise, OHIP-14 scores were not significantly associated with diabetes status. This finding was similar to studies by Irani et al., [8] and Allen et al., [19] who found that T2DM did not impact OHRQoL. Nevertheless, it was found that chronic periodontitis and gingivitis were associated with poorer OHRQoL in non-diabetic patients. One possible explanation for the lack of association between diabetes and OHRQoL, was that diabetic patients may have been more concerned about other health problems related to their diabetes. Therefore, they may not have prioritized oral health as having a significant impact on their well-being [8]. Furthermore, cultural differences in the interpretation of the impact of a disease on OHRQoL must also be considered [35]. Moreover, in this study, T2DM patients did not experience more decay than the non-diabetic control group, which may also explain the lack of association between diabetes status and OHRQoL. This is substantiated by the findings of the linear regression models in which DMFT scores were significantly associated with four out of seven OHIP-14 domains (physical disability, physical pain, psychological discomfort, and psychological disability), irrespective of diabetic status. The same four domains, in addition to the functional limitation domain, were associated with DMFT scores in non-diabetic patients. The observation that dental caries has a large impact on OHRQoL has also been shown in other studies [23]. Similarly to periodontal disease, the lack of a significant association between DMFT scores and OHRQoL among diabetic patients may have been because the overall burden of diabetes reduced the impact of oral health issues on quality of life.

There is evidence supporting the hypothesis that stress may have a role in the progression of periodontitis in susceptible patients, by modifying the host immune response [36]. Indeed, stress and depression are recognized as common psychosocial problems in diabetic patients that can modify the onset and progression of periodontitis [37]. This may explain our observation that CAL was significantly associated with both the social disability and handicap domains in diabetic patients, while only the handicap domain reached statistical significance among non-diabetic patients.

While this study provides evidence for an association between diabetes and oral health-related quality of life, the case-control study design does not allow firm conclusions to be drawn regarding causality. In addition, our study sample was comparatively young and had only been diagnosed with diabetes for a relatively short period of time. However, despite these limitations, the results of this study can be considered important baseline data for further studies in this area.

## Conclusions

Participants with decayed teeth reported substantial physical and psychological impacts on OHRQoL, irrespective of their diabetic status. Diabetic patients may be more likely to focus on other health issues compared to their oral health, which could explain the relative lack of impact of tooth decay on their OHRQoL. CAL may have a more severe impact on social disability among diabetic patients, while impacts on the handicap domain may be equally relevant in both diabetic and non-diabetic patients. There is a need for the development of preventive health care interventions that target improvements in quality of life, and such measures should be tailored to the treatment needs of the specific medically compromised patient population in question.

## Data Availability

The datasets generated and/or analyzed during the current study are not publicly available because it is not based on populations but a sample of patients, but are available from the corresponding author on reasonable request.

## Abbreviations

CAL: Clinical attachment loss
HRQoL: Health-related quality of life
OHIP-14: Oral Health Impact Profile short form
T2DM: Type 2 diabetes
